# Sweepoviruses Cause Disease in Sweet Potato and Related *Ipomoea* spp.: Fulfilling Koch's Postulates for a Divergent Group in the Genus *Begomovirus*

**DOI:** 10.1371/journal.pone.0027329

**Published:** 2011-11-02

**Authors:** Helena P. Trenado, Anelise F. Orílio, Belén Márquez-Martín, Enrique Moriones, Jesús Navas-Castillo

**Affiliations:** Instituto de Hortofruticultura Subtropical y Mediterránea “La Mayora” (IHSM), Consejo Superior de Investigaciones Científicas, Algarrobo-Costa, Málaga, Spain; Institute of Infectious Disease and Molecular Medicine, South Africa

## Abstract

Sweet potato (*Ipomoea batatas*) and related *Ipomoea* species are frequently infected by monopartite begomoviruses (genus *Begomovirus*, family *Geminiviridae*), known as sweepoviruses. Unlike other geminiviruses, the genomes of sweepoviruses have been recalcitrant to rendering infectious clones to date. Thus, Koch's postulates have not been fullfilled for any of the viruses in this group. Three novel species of sweepoviruses have recently been described in Spain: *Sweet potato leaf curl Lanzarote virus* (SPLCLaV), *Sweet potato leaf curl Spain virus* (SPLCSV) and *Sweet potato leaf curl Canary virus* (SPLCCaV). Here we describe the generation of the first infectious clone of an isolate (ES:MAL:BG30:06) of SPLCLaV. The clone consisted of a complete tandem dimeric viral genome in a binary vector. Successful infection by agroinoculation of several species of *Ipomoea* (including sweet potato) and *Nicotiana benthamiana* was confirmed by PCR, dot blot and Southern blot hybridization. Symptoms observed in infected plants consisted of leaf curl, yellowing, growth reduction and vein yellowing. Two varieties of sweet potato, ‘Beauregard’ and ‘Promesa’, were infected by agroinoculation, and symptoms of leaf curl and interveinal loss of purple colouration were observed, respectively. The virus present in agroinfected plants was readily transmitted by the whitefly *Bemisia tabaci* to *I. setosa* plants. The progeny virus population present in agroinfected *I. setosa* and sweet potato plants was isolated and identity to the original isolate was confirmed by sequencing. Therefore, Koch's postulates were fulfilled for the first time for a sweepovirus.

## Introduction

Geminiviruses are a diverse group of plant viruses with circular single-stranded DNA genomes that are characterized by their unique, geminate particle structure. Four genera have been described, which differ in genome organization, host range and insect vector [1]. Geminiviruses that are transmitted by the whitefly *Bemisia tabaci*, with either bipartite or monopartite genomes, are included in the genus *Begomovirus*. Sweet potato [*Ipomoea batatas* (L.) Lam.] (Convolvulaceae) is an important staple food crop which ranks as the seventh in terms of production [2]. It is vegetatively propagated and therefore prone to virus accumulation. Over 20 viruses are known to infect sweet potato, from the genera *Carlavirus, Nepovirus, Cucumovirus, Enamovirus, Tospovirus, Ipomovirus, Potyvirus, Crinivirus, Begomovirus* and *Cavemovirus* [3], [4].

Sweet potato leaf curl disease was first reported in Taiwan and Japan [5], [6], [7], and a similar disease was also observed in Israel [8]. These reports indicated that the causal agents were whitefly-transmitted geminiviruses. The first completely sequenced begomovirus infecting sweet potato was *Sweet potato leaf curl virus* (SPLCV), described in the USA [9], [10], [11]. To date, sweet potato leaf curl virus and related viruses have been found in many other countries, including Peru, Italy, Spain, China, Korea, Kenya, Uganda, India and Brazil [12], [13], [14], [15], [16], [17], [18], [19], [20], [21], [22], [23]. The name sweepoviruses has been suggested for the sweet potato begomoviruses [24]. This unique group clusters separately from the rest of the begomovirus species and appears to belong to a branch distinct from the Old and New World groups. This branch seems to represent one of the earliest points of divergence within this genus [16]. Several reports have described substantial yield losses in some sweet potato cultivars due to sweepoviruses [25], [26].

Obtaining infectious begomovirus clones is a relatively easy process that has been simplified in various ways following the development of the rolling circle amplification (RCA) methodology using φ29 DNA polymerase [27], [28], [29]. Nevertheless, the genomes of sweepoviruses have been recalcitrant to rendering infectious clones to date [10], [21]. Thus, Koch's postulates have not yet been fulfilled for any of the viruses in this group.

Three novel species of sweepoviruses have recently been described in Spain: *Sweet potato leaf curl Lanzarote virus* (SPLCLaV), *Sweet potato leaf curl Spain virus* (SPLCSV) and *Sweet potato leaf curl Canary virus* (SPLCCaV). SPLCLaV was first isolated from a sweet potato plant from Lanzarote (Canary Islands, Spain) and later found in the Malaga province of continental Spain [16]. In this investigation we report the construction of a complete tandem dimer genome clone of an isolate of SPLCLaV (ES:MAL:BG30:06) after partial digestion of an RCA product. The clone was successfully agroinoculated to *Nicotiana benthamiana*, *I. nil*, *I. setosa*, and sweet potato ‘Beauregard’ and ‘Promesa’ plants, which became infected. The virus present in agroinfected plants was readily transmitted by the whitefly *B. tabaci* biotypes B and Q. Disease symptoms similar to those observed in nature occurred in inoculated sweet potato plants and the progeny of the viral clone was confirmed to be identical to the original isolate. Therefore, Koch's postulates were fulfilled for the first time for a sweepovirus.

## Results

### A dimeric SPLCLaV clone is infectious to *N. benthamiana*, sweet potato and other *Ipomoea* species by agroinoculation

**Figure 1:**
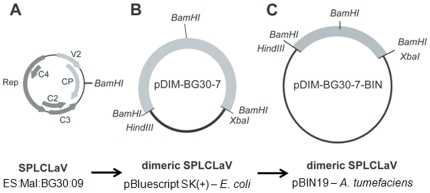
Strategy to generate the infectious dimeric clone of SPLCLaV, pDIM-BG30-7-BIN. (A) Genetic map of SPLCLaV, isolate ES:Mal:BG30:09, showing the *Bam*HI restriction site used to generate a tandem dimeric clone after partial digestion of the RCA product obtained from an infected plant. (B) Dimeric SPLCLaV cloned in pBluescript SK(+) in *E. coli* (pDIM-BG30-7). (C) Dimeric SPLCLaV cloned in the binary vector pBIN19 in *A. tumefaciens* (pDIM-BG30-7-BIN).

**Figure 2:**
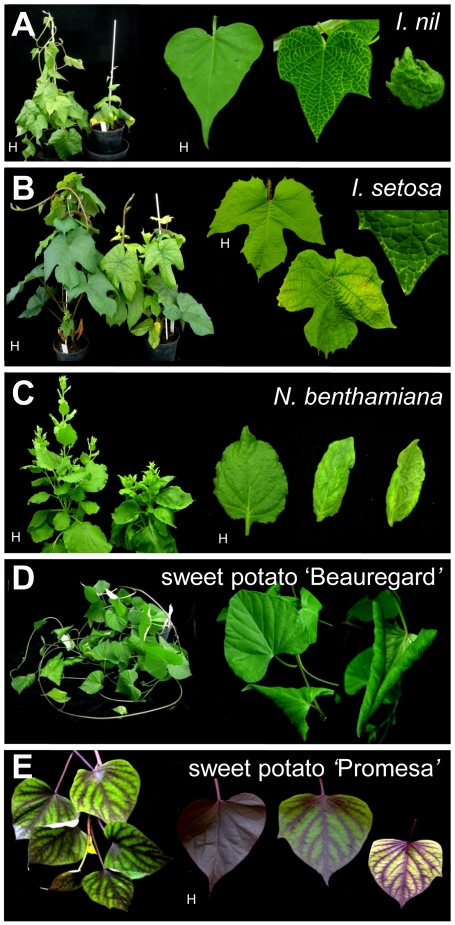
Symptoms observed in plants agroinfected with the clone pDIM-BG30-7-BIN of SPLCLaV. (A) Growth reduction, leaf curling and vein yellowing in *I. nil*. (B) Growth reduction, yellowing, leaf curling and vein yellowing in *I. setosa*. (C) Growth reduction, yellowing and leaf curling in *N. benthamiana*. (D) Leaf curling in sweet potato cv. ‘Beauregard’. (E) Loss of purple pigmentation in sweet potato cv. ‘Promesa’. Symptoms were recorded at 35 days (A–C) and 5 months (D, E) post-inoculation. H, healthy control plants.

**Figure 3:**
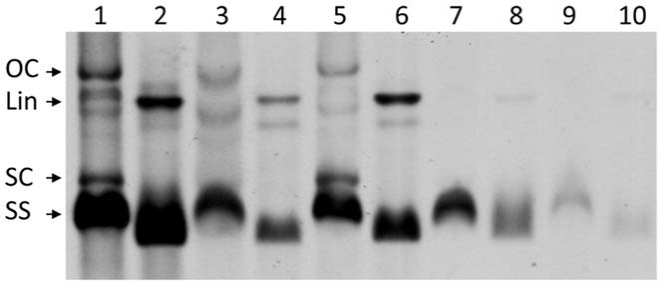
Southern blot analysis of SPLCLaV infected plants. DNA was isolated from *I. nil* (lanes 1–2), *N. benthamiana* (lanes 3–4), *I. setosa* (lanes 5–6) and sweet potato cv. ‘Beauregard’ (lanes 7–8) plants agroinfected with pDIM-BG30-7-BIN, and *I. purpurea* (lane 9–10) plant infected by grafting. Lanes 2, 4, 6, 8 and 10 correspond to DNA digested with *Bam*HI. Positions are indicated for the single-stranded genomic DNA (ss) and double-stranded replicative DNA forms [supercoiled (SC), linear (Lin) and open circular (OC)].

Following the methodology developed by Ferreira et al. [28] to clone begomovirus genomes following partial digestion of RCA products, we obtained a tandem dimeric clone in pBluescript SK(+) corresponding to the genome of SPLCLaV, isolate ES:MAL:BG30:06 (BG30), which was designated pDIM-BG30-7. Complete sequencing of the insert of this clone showed 100% nucleotide identity with the original BG30 isolate [16]. The insert was subcloned into the binary vector pBIN19, to render pDIM-BG30-7-BIN (Fig. 1), which was transformed in *A. tumefaciens* strains LBA4404 and GV3103. In preliminary experiments, stems and cotyledons of *I. nil* and *I. setosa* were agroinoculated using strain LBA4404 and, although no symptoms were observed in the inoculated plants, some of the *I. setosa* plants became infected, as determined by PCR (Table 1). One of the agroinfected *I. setosa* plants was used as a source of inoculum in successful whitefly transmission experiments using *B. tabaci* (see below). Agroinoculation experiments, carried out with strain GV3103 and various plant species, resulted in successful infection by SPLCLaV of *N. benthamiana*, *I. setosa*, *I. nil*, and sweet potato ‘Beauregard’ and ‘Promesa’ plants. In this case, at 21 days post-inoculation (dpi), *I. nil* and *I. setosa* plants developed leaf deformation including curling, yellowing and growth reduction, while sweet potato and N. benthamiana plants remained asymptomatic. At late infection stages (∼35 dpi), *I. nil* and *I. setosa* plants showed vein yellowing and *N. benthamiana* plants showed yellowing, leaf curling and growth reduction (Fig. 2), but sweet potato remained asymptomatic. The symptoms in *I. nil* were more consistent and conspicuous than those in *I. setosa*. Symptoms of leaf curling in ‘Beauregard’ and the interveinal loss of purple pigmentation in ‘Promesa’ were sporadically observed when plants were kept in the growth chamber for 4–6 months post-inoculation (Fig. 2). None of the agroinoculated *I. purpurea* plants became infected with SPLCLaV. Infection in all the aforementioned hosts, including asymptomatic sweet potato plants, was confirmed by PCR and dot blot hybridization. In addition, Southern blot analysis using a probe to the CP gene of the BG30 isolate revealed the presence of the characteristic viral forms of begomovirus infection in agroinoculated *I. nil*, *I. setosa*, sweet potato ‘Beauregard’ and *N. benthamiana* plants, i.e., single-stranded genomic DNA and double-stranded replicative DNA forms (supercoiled, linear and open circular) (Fig. 3). None of the agroinoculated *I. purpurea* plants became infected. In an attempt to infect *I. purpurea* with the BG30 isolate, vines of this species were grafted with scions of agroinfected *I. nil* plants; although no symptoms were observed, two out of five grafted *I. purpurea* plants became infected with SPLCLaV, as shown by Southern blot analysis (Fig. 3).

**Table 1:** Transmission experiments with SPLCLaV.

Inoculation method		Plant host					
						Sweet potato	
		*N. benthamiana*	*I. nil*	*I. setosa*	*I. purpurea*	‘Beauregard’	‘Promesa’
Agroinoculation^a^	LBA4404	-	0/10^c^	3/10	-	-	-
	GV3103	9/9	7/7	10/10	0/15	8/15	5/10
*B. tabaci* b	Q	-	6/9	12/15	-	0/14	1/10
	B	-	2/4	3/4	-	0/4	-
	S	-	0/4	0/14	-	0/14	0/10
Grafting		-	-	-	2/5	-	-

a *A. tumefaciens* strains are indicated.

b *B. tabaci* biotypes are indicated.

c Figures correspond to number of infected plants per inoculated plants for each plant host and inoculation method. Virus detection was performed by dot-blot molecular hybridization using a CP gene probe and/or PCR with specific primers designed to the same gene. -, not done.

### SPLCLaV isolate BG30 is transmissible by *B. tabaci*

To test the transmissibility of the cloned SPLCLaV isolate by *B. tabaci* biotype Q, an experiment was carried out using one agroinfected *I. setosa* plant. The progeny of the infectious SPLCLaV clone present in this plant was readily transmitted to *I. nil* and *I. setosa* test plants (Table 1). This was confirmed by the observation of symptoms (growth reduction, leaf curling and vein yellowing) and PCR. Additional experiments conducted with different plant hosts using different *B. tabaci biotypes* (Table 1) showed that both the Q and B biotypes could readily transmit SPLCLaV to *I. nil* and *I. setosa*, and one of 10 sweet potato ‘Promesa’ test plants could be infected using the Q biotype. None of the sweet potato ‘Beauregard’ and *I. purpurea* plants could be infected either using the Q or B biotype.Transmission did not occur when *B. tabaci* biotype S was used.

### The SPLCLaV progeny population generated in *I. setosa* and sweet potato agroinfected plants is identical to the original isolate

The SPLCLaV progeny population present in an agroinfected sweet potato ‘Beauregard’ plant and in a whitefly-infected *I. setosa* plant was analyzed by sequencing clones derived after RCA amplification. Sequence analysis confirmed the 100% nucleotide identity of the virus present in the sweet potato plant with the original cloned isolate, whereas the virus present in the *I. setosa* plant contained only one nucleotide change at nucleotide 309 (G to T), which introduced an amino acid change at position 89 in the V2 gene (tryptophan to cysteine).

## Discussion

The availability of infectious clones for plant viruses has proved to be a powerful molecular tool for studying aspects of their biology, being essential for functional analysis of viral genes and their role in replication, pathogenesis and transmission. Infectious clones have also been used to facilitate the screening of germplasm for virus resistance. The agroinoculation of viral clones using binary vectors is a highly efficient process, and is widely used to infect plants with begomoviruses and other members of the family *Geminiviridae* [30], [31]. Although it has been demonstrated that a clone containing a single genome copy is sufficient to ensure infectivity for certain begomoviruses [32], [33], [34], [35], the use of clones with duplicate copies of the sequences involved in the initiation of replication generally increases infectivity [31], [33], [36], [37], [38], [39], [40], [41]. Recently, a simple method for the construction of infectious begomovirus clones has been developed by cloning dimeric forms of the genome in a binary vector following partial digestion of the DNA obtained after rolling circle amplification with phage φ29 DNA polymerase [28], [29]. Despite the availability of relatively simple methodology for generating infectious clones of begomoviruses, which has been successfully applied to a large number of species, infectious clones were not obtained for any of the monopartite begomoviruses that infect *Ipomoea* spp., known as sweepoviruses. Therefore, Koch's postulates could not be demonstrated for observed diseases putatively attributed to these viruses. To our knowledge, at least two unsuccessful attempts to achieve infectivity from cloned sweepovirus DNA have been reported. Thus, the cloned genomic DNA of *Sweet potato leaf curl Georgia virus* (formerly Ipomoea leaf curl virus) obtained after excision from the plasmid vector and in vitro circularization, was not able to infect any of the 27 *I. nil* plants inoculated using electric discharge particle acceleration [10]. Similarly, infection was not achieved in biolistics experiments using RCA-amplified multimers of cloned DNA from several sweepoviruses from Brazil, assayed in *N. benthamiana* and several *Ipomoea* species [21]. In this work, a dimeric clone of a sweepovirus recently described in Spain, *Sweet potato leaf curl Lanzarote virus* [16], was constructed following rolling circle amplification of the genome DNA [27], partial digestion [28], and cloning in a standard binary vector. The resulting clone was shown to be infectious in *N. benthamiana*, *I. nil*, *I. setosa* and sweet potato ‘Beauregard’ and ‘Promesa’ plants, and symptoms including leaf curling, yellowing, growth reduction, vein yellowing, and interveinal loss of purple colouration (in the case of ‘Promesa’) were observed in infected plants. Symptoms of vein yellowing and leaf curling have been reported for sweet potato under natural infections in plants from which sweepoviruses such as SPLCV [9], [11] and *Ipomoea yellow vein virus* (IYVV) [14], [15] were isolated. In previous studies, leaf symptoms were not observed in ‘Beauregard’ sweet potato plants naturally infected with SPLCV [9], [10], [25], although yield losses were associated with infection by this virus. Clark and Hoy [25] illustrated the potential importance of SPLCV infection in a field study carried out in Louisiana, USA, where they found a yield reduction of 25–30% in the cultivar ‘Beauregard’ when infected with this virus. Recently, similar results have been reported for ‘Beauregard’ and other cultivars in a field trial in South Carolina, USA, with a reduction in the average number of roots and the percentage of US#1 class roots per plant in the SPLCV-infected plants [26]. However, direct cause-effect for symptoms or yield losses associated with sweepovirus infection could not be demonstrated experimentally because infectious clones were not available. In preliminary experiments carried out with the SPLCLaV infectious clone (our unpublished results), although no significant difference was observed in total yield, SPLCLaV-infected sweet potato ‘Beauregard’ plants reduced the yield of Jumbo plus US#1 class roots, whereas they increased the yield of Large canner and Small canner classes, which are smaller and have less commercial value. Confirmation of these results would reinforce the need to control sweepovirus infection in sweet potato by incorporating specific diagnosis tests into sweet potato virus indexing protocols The practice carried out by farmers in some countries, whereby they select symptomless vine cuttings to propagate material from genotypes that have virus resistance, or rogue symptomatic plants from fields in an attempt to reduce viral disease [42], [43], can be compromised by asymptomatic sweepovirus infections [3].

The progeny of the infectious SPLCLaV clone was successfully transmitted by *B. tabaci* biotype Q and B from agroinfected I. setosa plants to healthy *I. nil*, *I. setosa* and sweet potato “Promesa” plants, demonstrating that a fully biologically active genome molecule was cloned. Although transmission assays were conducted with 40–50 whiteflies per plant, the transmission rate to sweet potato plants was very low, as found in other studies. It has been suggested that such low transmission rates may be a reflection of the low amino acid sequence identity between the coat protein of sweepoviruses and those of other begomoviruses [44]. SPLCV isolates from Taiwan and Japan were transmitted by *B. tabaci* only when a high number of insects were used per plant [5], [7]. In contrast, high rates of transmission (50–60%) of a sweepovirus isolate associated with Ipomoea crinkle leaf disease in Israel were obtained with batches of 50 whiteflies per test plant, whereas the transmission efficiency was 20–30% when using 10 whiteflies per plant [45]. Simmons et al. [46] suggested that females may be more efficient than males in vectoring SPLCV and that the efficiency of *B. tabaci* in acquiring and transmitting SPLCV to sweet potato is lower than in most other host–whitefly systems. On the other hand, the unsuccessful transmission of IYVV to *I. indica* using the Q, B, and S *B. tabaci* biotypes, led to the suggestion that in some cases sweepovirus could have lost the capacity for transmission in nature due to the continued vegetative propagation of its natural host plant [15].

In conclusion, the results of this investigation demonstrated that a sweepovirus clone was infectious by agroinoculation and induced symptoms in *N. benthamiana*, *I. nil*, *I. setosa* and two cultivars of sweet potato, ‘Beauregard’ and ‘Promesa’. The cloned virus was also transmissible by two biotypes of *B. tabaci*, and the viral progenies present in infected *I. setosa* and sweet potato plants were identical to the originally inoculated virus. Symptoms similar to those observed in naturally infected sweet potato plants were observed in experimentally infected plants, thus Koch's postulates were fulfilled for the first time for a disease caused by a sweepovirus belonging to a unique divergent group of the genus *Begomovirus* infecting sweet potato and other *Ipomoea* spp.

## Materials and Methods

### Virus source

The isolate of *Sweet potato leaf curl Lanzarote virus* (SPLCLaV) used in this study, ES:Mal:BG30:09 (BG30), was obtained from a sweet potato plant growing at Algarrobo (Málaga province, southern Spain) in 2006 and has since been maintained in our laboratory by periodical transmission by *B. tabaci* biotype Q in *I. nil*. The inoculated *I. nil* plant showed vein yellowing and leaf curling symptoms. The complete nucleotide sequence of isolate BG30 has been described previously [16].

### Production of a dimeric clone

To generate dimeric clones of isolate BG30, we started from the rolling circle amplification (RCA) product obtained from the aforementioned *I. nil* plant and previously used for cloning and sequencing of that isolate [16]. The RCA product was used to generate a complete tandem dimer of the viral genome using the partial digestion method described by Ferreira *et al*. [28]. Briefly, about 6 µg of amplified DNA was digested with 4 U of *Bam*HI, which cut at a single site in the viral genome, in a final volume of 60 µL at 37°C for 10 min. The DNA band corresponding in size to a dimer of the viral genome (∼5.6 Kbp) was recovered from a 0.8% agarose gel after electrophoresis. The recovered fragment was purified using the DNA Gel Extraction System (Millipore). The purified dimeric DNA fragment was ligated into the vector pBluescript SK(+) (Stratagene) previously digested with *Bam*HI and dephosphorylated with shrimp alkaline phosphatase (Roche). The ligation product was transformed into *Escherichia coli* DH5α cells by electroporation. A clone, designated pDIM-BG30-7, was selected and completely sequenced in an ABI 3700 automatic sequencer (Secugen, Madrid, Spain). The insert of the clone pDIM-BG30-7 was released by digestion with *Hind*III and *Xba*I and subcloned in the binary vector pBIN19. Clone pDIM-BG30-7-BIN, shown to contain a dimeric complete viral genome of isolate ES:Mal:BG30:09, was selected (Figure 1).

### Agroinoculation

Clone pDIM-BG30-7-BIN was transferred to *Agrobacterium tumefaciens* strains LBA4404 and GV3101 by electroporation. Cultivated bacteria were resuspended in induction buffer (10 mM MES pH 5.8, 10 mM MgCl_2_, 150 µM acetosyringone) to obtain OD_600_ = 1. Plant were agroinoculated in the stem using a syringe with a 25 GA needle and agroinfiltrated into the leaves using the same syringe after removing the needle. Seedlings of *I. nil*, *I. setosa* and *I. purpurea* were agroinoculated at the cotyledon stage and seedlings of *N. benthamiana* at the 5 leaf stage. Sweet potato cuttings were agroinoculated when they had 2–3 newly developed leaves. Plants were kept in a growth chamber or an insect-free greenhouse for about 45 days and were usually tested for virus infection at 20 and 40 dpi.

### Plant DNA extraction

Leaf samples (50–100 mg) were ground in the presence of 500 µL of extraction buffer (100 mM Tris-HCl, 20 mM EDTA, 1.4 M NaCl, 2% CTAB, 0.5 M glucose, 100 mM DTT, 2 mM β-mercaptoethanol, pH 8) at a speed set of 4.0 for 40 s in a FastPrep-24 homogenizer (MP Biomedicals). The homogenate was shaken at 200 rpm at 60°C for 1 h. Following the addition of 500 µL of chloroform:isoamyl (24∶1), the homogenate was shaken again at 200 rpm at room temperature for 5 min. After centrifugation at 20000 g at 4°C for 5 min, the supernatant (∼ 400 µL) was recovered, mixed with 0.8 volumes of isopropanol and centrifuged at 20000 g at 4°C for 10 min. The precipitated DNA was washed with 70% ethanol, dried, resuspended in 100 µL of water, and kept at −20°C until analysis.

### Grafting inoculation

SPLCLaV isolate BG30 was transmitted by grafting vine segments of an agroinfected *I. nil* plant onto healthy *I. purpurea* seedlings.

### Detection of SPLCLaV isolate BG30

The presence of SPLCLaV isolate BG30 DNA in inoculated plants was assessed by dot blot hybridization and/or polymerase chain reaction (PCR), typically at 20 and 40 dpi. For dot blot hybridization, 1 µL of total DNA extracted from inoculated plants was applied to positively charged nylon membranes (Roche) and hybridized with a digoxigenin-labeled probe synthesized by PCR from clone pDIM-BG30-7. The insert was released from the vector by digestion with *Pst*I and *Xba*I and used to amplify the coat protein (CP) gene by PCR with primers MA292 (+) (5′- CCYTAGGGTTCGAGCTVTGTTCGG -3′) and MA293 (-) (5′- TTTATTAATTDTTRTGCGAATC-3′) [16] incorporating DIG-dUTP with the PCR DIG Probe Synthesis System Kit (Roche) according to the manufacturer's instructions. PCR detection was performed with primers MA292 and MA293 as previously described [16]. For Southern blot analysis, total DNA from inoculated plants (∼5 µg) was digested with *Bam*HI, and separated by 0.8% agarose gel electrophoresis in TBE containing ethidium bromide for 3 h at 50 V. After depurination, denaturation and neutralization steps, DNA was transferred to a positively charged nylon membrane (Roche) by applying a vacuum (VacuGene XL, HealthCare) and fixed with ultraviolet light treatment (Crosslinker RPN 2500, Amersham Life Science). DNA was then hybridized with the aforementioned CP probe.

### Whitefly transmission of the virus progeny from agroinfected plants

Transmission experiments were carried out using *B. tabaci* biotypes Q, B and S from healthy populations maintained in insect-proof screen cages (Table 1). The Q and B biotypes were reared on melon (*Cucumis melo* cv. ANC 42, La Mayora-CSIC seed bank) plants and the S biotype was reared on *I. nil* plants. In each experiment, about 2000 adult insects were released into a cage containing a SPLCLaV-agroinfected *I. setosa* plant, where they fed freely for 48 h. Then, they were transferred in groups of 40–50 to clamp cages (one per plant) and placed on healthy *I. setosa*, *I. nil* and sweet potato plants. *I. nil* and *I. setosa* plants were inoculated at the cotyledon stage and sweet potato plants were inoculated as cuttings with 2–3 newly developed leaves. After a transmission period of 48 h, the plants were sprayed with Confidor 20 LS (Imidacloprid 20 %) and Atominal 10 EC (Pyriproxyfen 10 %) and kept in a growth chamber or an insect-proof greenhouse and monitored for symptoms.

### Characterization of the progeny begomovirus

Total DNA was isolated from two selected samples each consisting of 100 mg of symptomatic leaf tissue from *I. setosa* and sweet potato ‘Beauregard’ using the CTAB-based method described above. The *I. setosa* plant was inoculated with *B. tabaci* from an *I. setosa* plant agroinfected using strain LBA4404, and the sweet potato ‘Beauregard’ plant was agroinfected using strain GV3101. Amplification of begomovirus genome DNA was performed using rolling circle amplification (RCA) with φ29 DNA polymerase (TempliPhi kit, GE Healthcare). A 2814-bp fragment, corresponding to the full-length genome, was obtained from both samples by digestion of the RCA product with *Bam*HI and cloned in pBlueScriptII SK+ (Stratagene). One clone per sample was selected and sequenced (Macrogen Inc., Seoul, South Korea). Sequence data were analysed using SeqMan and SeqBuilder and the nucleotide sequence identity was calculated using MegAlign based on the multiple sequence alignment obtained with Clustal V using default parameters (DNASTAR package, DNASTAR Inc.).
